# Tuberculosis-Human Immunodeficiency Virus (HIV) co-infection in Ethiopia: a systematic review and meta-analysis

**DOI:** 10.1186/s12879-018-3604-9

**Published:** 2018-12-18

**Authors:** Mebrahtu Teweldemedhin, Negasi Asres, Hailay Gebreyesus, Solomon Weldegebreal Asgedom

**Affiliations:** 1 Department of Medical Laboratory Sciences, College of Health Sciences, Aksum University, PO.BOX 298, Aksum, Tigray Ethiopia; 2 Department of Public Health, College of Health Sciences, Aksum University, Aksum, Tigray Ethiopia; 3 School of Pharmacy, College of Health Sciences, Mekelle University, Mekelle, Tigray Ethiopia

**Keywords:** Tuberculosis, Human immunodeficiency virus, Co-infection, Prevalence, Meta-analysis

## Abstract

**Background:**

Human Immunodeficiency Virus (HIV) and Tuberculosis (TB) are the double burden diseases of the world. The African continent takes a great share of TB-HIV cases worldwide. This study was aimed to determine the prevalence of TB-HIV co-infection in Ethiopia, using a meta-analysis based on a systematic review of published articles.

**Methods:**

An electronic search was conducted in databases including PubMed, HINARI, EMBASE, Cochrane library and Google Scholar to extract the articles. Articles published between 1995 and November 2017 had been searched for using different keywords. The analysis was performed using MetaXL software and R statistical software (version 3.2.3).

**Result:**

Our searches returned a total of (*n* = 26,746) records from 30 articles of which 21 were cross-sectional, 7 were retrospectives and 2 were prospective studies. The range of prevalence of TB-HIV co-infection was found to be from 6 to 52.1% with random effects pooled prevalence of 22% (95% CI 19–24%) and with substantial heterogeneity chi-square (X^2^*) =* 746.0, *p* < 0.001, (*I*^*2*^ *= 95.84%*).

**Conclusion:**

Our analysis indicated that the prevalence of TB-HIV co-infection is high in Ethiopia with substantial regional variation. An integrated, facility-based and community-based effort towards the prevention, early detection and management of cases should be further strengthened throughout the country to mitigate the double burden disease.

## Background

Human Immunodeficiency Virus (HIV) remained as the leading cause of mortality and morbidity; in 2016 for instance, 1.8 million people were newly infected and 1 million AIDS-related deaths were registered [1]. HIV/AIDS and TB (Tuberculosis) are considered as the double burden diseases of the world. According to the World health organization (WHO) report, there were 1.5 million deaths attributed to TB out of which 26% were due to HIV-associated TB [2]. In developing countries, particularly in sub-Saharan Africa, TB is increasing due to the burden of HIV [3]. Hence, the African continent takes the greater share (74%) of the 1.2 million TB-HIV cases worldwide [2]. In Ethiopia, 4 in 100 people die due to TB-HIV co-infection and the incidence of Multidrug-resistant Tuberculosis (MDR-TB) was estimated to be 5.8 per 1000 people [4].

The burden of TB is still alarming due to the emergences of drug-resistant strains. Recent studies indicated that the prevalence of MDR-TB in Ethiopia is particularly noticeable (15%) in previously treated TB cases [5]. Among laboratory-confirmed TB cases in the Oromo region, for instance, 33% of the cases were found to be MDR-TB, majorly among the adult population [6]. This increase in MDR-TB, especially among people living with HIV is a great burden to the healthcare system; challenging the management efforts of the disease through drug interaction and immune reaction [7–9]. The associated morbidity and mortality related to noticeable economic loss of the productive society and the socioeconomic status of the country at large. In developing countries, it is estimated that around 30% of the annual household income is taken by TB and HIV associated disease [10].

In general, people living with HIV are 20 times riskier to be infected with TB as compared to HIV negative people [11], with case fatality rate of 16–35%, which is almost 4 times higher than HIV free individuals [12]. The incidence of co-infection is determined by many factors including smoking, family size, the clinical stage of HIV, use of antiretroviral therapy (ART), injectable drug use and anemia [13–15]. In addition, it is evident that HIV positivity, alcohol intake, farming occupation and previous TB contact are contributing factors for MDR-TB [6].TB and HIV co-infection has various health outcomes limiting the life expectancy of the infected host; accelerated by malnutrition, rapid progression of the disease, atypical TB presentations, delayed diagnosis and a low treatment response [16]. Active tuberculosis facilitates the transcription of HIV genes resulting in increased diversity and replication of HIV [17]. On the other hand, as the clinical stage of HIV progresses, there is an increased dissemination and extra-pulmonary TB manifestations [18]. Finally, patients suffer due to frequent pyrexia, diarrhea, severe weight loss and wasting [19, 20].

In high burden and resource-limited countries like Ethiopia, integrating health services on TB and HIV has paramount importance in order to increase the effectiveness of the diagnosis and to improve the management approaches [21]. Considering this, the WHO introduced collaborative strategies to control HIV associated TB and Ethiopia is one of the countries exercising this program as part of the health care system [22]. The Centers for Disease Control (CDC) program in Ethiopia (opened in 2001) is providing guidance and technical support towards TB-HIV co-infection and MDR-TB services. As a result, large improvements have been achieved in HIV testing, ART provision and diagnostic potential. Studying the prevalence of TB-HIV co-infection is important for programmatic changes or further improvements on the existing programs. Therefore, this study was aimed to determine the prevalence of TB-HIV co-infection in Ethiopia, using a meta-analysis based on a systematic review of published articles.

## Methods

### Search strategy and inclusion criteria

An electronic search was conducted to retrieve and recruit studies. Published articles of cross-sectional, prospective and retrospective cohort studies were included, which focused on the “Prevalence of TB-HIV co-infection in Ethiopia”. Databases PubMed, HINARI, EMBASE, Cochrane library, and Google Scholar search engine were used to extract journals. Articles published between 1995 and November 2017 had been searched for and their full-text was retrieved by two independent reviewers.

#### Inclusion criteria

The predetermined inclusion criteria for this analysis were: 1) studies conducted in Ethiopia, 2) either longitudinal/cohort or cross-sectional studies which assessed the prevalence of TB/HIV co-infection in Ethiopia and has a sample size of greater than 100 (*n* > 100). 3) Studies written in English. 4) Articles which were published and available online.

**Fig. 1:**
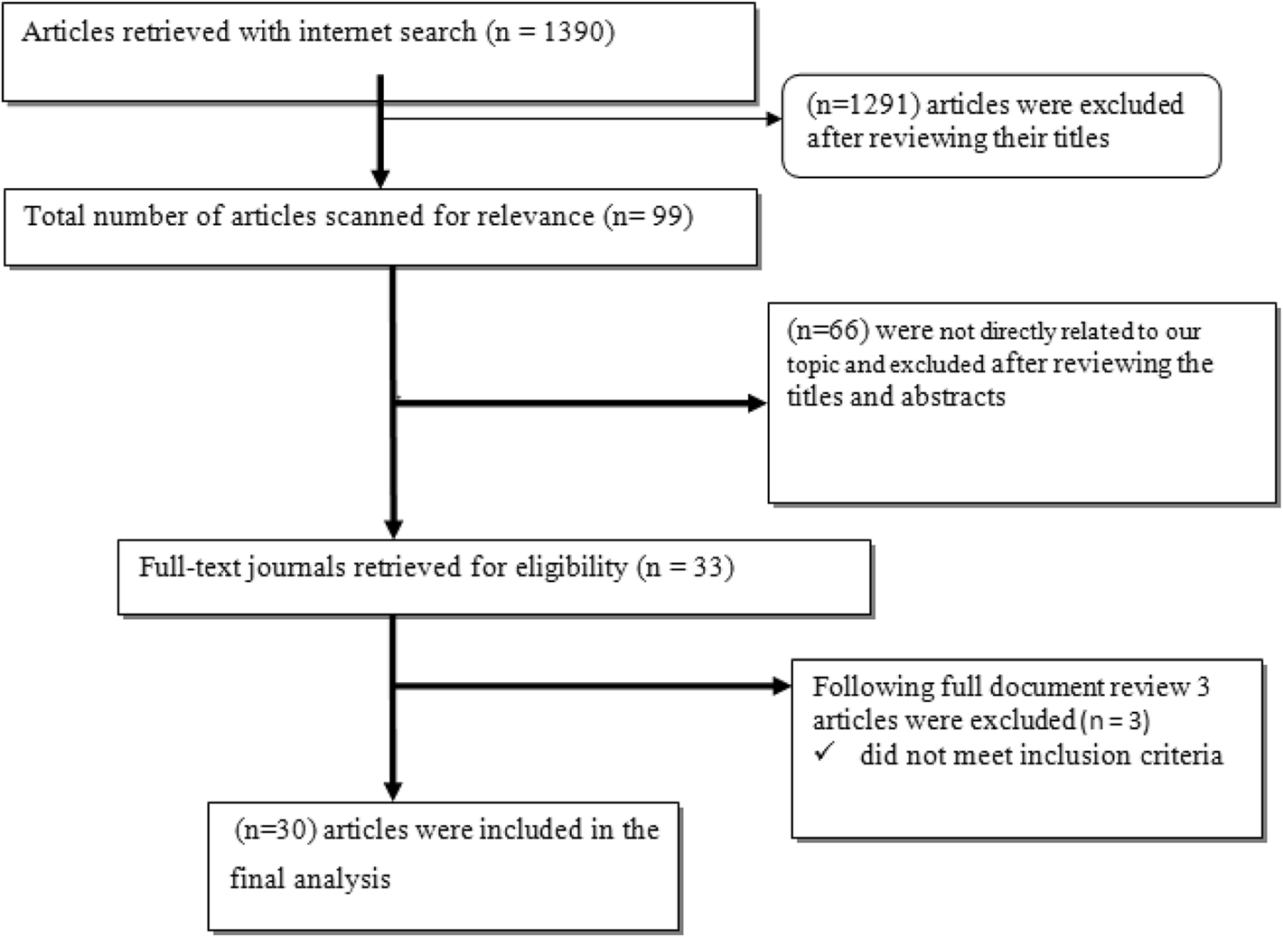
Flow diagram of articles selection process

Based on these inclusion criteria, 30 articles were selected and analyzed (Fig. 1).

### Data extraction and synthesis

We developed a data collection tool to collect the findings from the published articles. The data collection tool was pretested on 5 articles and an amendment was done accordingly. After the preliminary data extraction, a careful analysis was made to record the type of the study, study setting/region, author and year of publication, number of patients/participants and the main outcomes of the study on the data collection tool.

### Description of studies

As summarized in Fig. 1, the literature search with the chosen search terms firstly identified 1390 citations. We repossess 99 articles after reviewing the proximity of the title to the predetermined objectives, and after further reading the abstracts of each article, only 33 of the articles were subjected to document review. Lastly, 30 articles met the inclusion criteria for this systematic review and the meta-analysis (Fig. 1).

### Statistical analysis

We performed all analyses in Meta-XL software and R statistical software (version 3.2.3). When working on binary data with low prevalence, the inverse variance weight in fixed-effects meta-analyses is sub-optimum. Hence, the estimated prevalence was determined using the variance stabilizing double arcsine transformation [23]. Wilson method was used to calculate the 95% confidence intervals; the Cochran’s Q and *I*^*2*^ statistic were used to determine heterogeneity [24–26]. The I^2^ does not essentially depend on the number of studies included. Therefore, a high, moderate and low degree of heterogeneity indicates an *I*^*2*^ value of 75, 50, and 25% respectively. In this study, the *I*^*2*^ was greater than 75% and hence the summary statistics were made using the random-effects models in which the individual study weight is the sum of the weight used in the fixed effects model and the between-study variability [27]. To analyze the basis of heterogeneity, we performed a sensitivity analysis by excluding one largest study [28] and by analyzing the groups according to the geographic area.

## Results

### Study characteristics

Our searches returned a total of (*n* = 26,746) records from 30 articles of which 21 were cross-sectional, 7 were retrospectives and 2 were prospective studies. Furthermore, 17 studies had more than 500 participants; 12 studies were conducted in the Amhara region, 6 in southern Ethiopia, 2 in Tigray, 3 in Oromia, 3 in Addis Ababa, 2 in Afar and 2 across Ethiopia.

Nineteen [19] studies determined HIV infection among TB patients (*n* = 21,376) and 11 studies determined TB among HIV/AIDS patients (5370) (see Table 1).

**Table 1:** Summary of the studies in the review

Author, Year, (Ref.)	Study setting/region	Study design	Study population/participants	Prev. of TB-HIV co-infection
Alemayehu et al., 2014 [29]	Amhara Region, Gondar University; Hospital based	Cross-sectional	HIV patients, aged 18 years and above, *n* = 250	6%
Alemie & Gebreselassie, 2014 [30]	Amhara Region; selected private health institutions	Cross-sectional	TB patients, *n* = 1153	20%
Alemu, etal,2016 [31]	Amhara Region, West Gojjam and South Gondar, Population based	Retrospective	HIV patients, children aged less than 15, *n* = 645	12%
Ali et al.,2016 [32]	Across Ethiopia, Population based	Cross- sectional	HIV patients, aged 15–90, *n* = 575	29.4%
Balcha et al.,2014 [33]	Oromia Region, Adama; Population based	Prospective	HIV-patients, aged 18 years and above, *n* = 812	17.9%
Belay et al.,2015 [34]	Afar and Dessie Town; in 5 health facilities.	Cross-sectional	TB patients, aged above 18 *n* = 110	40%
Datiko et al.,2008 [35]	Southern Ethiopia, heath facilities	Cross-sectional	TB patients, *n* = 1261,	18%
Dengetu & dolamo, 2014 [36]	Addis Ababa; governmental health facilities	Facility-based Cross-sectional	TB patients, children aged 15–17 years, *n* = 579	35%
Deribew, et al.,2011 [37]	Addis Ababa; primary health care units	Facility based cross-sectional	TB patients, *n* = 298	27.2%
Fanosie et al.,2016 [38]	Amhara Region, University of Gondar Hospital	Institution based cross-sectional	TB patients, *n* = 141	14.1%
Fekadu et al., 2015 [39]	South Ethiopia	Retrospective	HIV/AIDS patients, *n* = 499	18.2%)
Belay et al., 2014 [40]	Afar region	Cross sectional	TB patients, *n* = 287	28.6%
Taddesse et al., 2013 [41]	Amhara region	Cross sectional	TB patients, *n* = 301	18.3%
Gebrecherkos et al., 2016 [42]	Amhara Region, North Gondar zone	Cross-sectional	TB patients, *n* = 282	27%
Gebreegziabiher et al., 2017 [28]	Tigray Region, Mekelle, Public health institutions	Cross-sectional	TB pregnant mother, *n* = 201	21.8%
Gebremariam, et al., 2016 [43]	Southern Rgion, Arsi Negele Health Center	Retrospective	TB patients, *n* = 1562	10%
Kassu et al.,2007 [44]	Amhara, Gondar University	Cross-sectional	TB patients, *n* = 257,	52.1%
Kebede et al., 2014 [45]	Oromiya, Este welega	Cross- sectional	TB patients, *n* = 406	33.7%
Kifle et al., 2014 [46]	Amhara, Northwest Shewa	Cross- sectional	TB patients, *n* = 335	28.7%
Ligidi et al.,2011 [47]	Oremiya, Adama Hospital based	Cross- sectional	TB patients, *n* = 258	26.4%
Mekonnen et al.,2015 [48]	Tigray Rgion,Southern zone	Retrospective	TB patients, *n* = 973	24.3%
Mihret et al., 2014 [49]	Addis Ababa Ethiopia	Cross-sectional	TB patients, *n* = 418	23.2%
Mitku et al. 2016 [50]	Amhara Region, Hospital based	Retrospective	HIV positive, *n* = 571	27.7%
Skogmar et al.,2014 [51]	Across Ethiopia	Cross-sectional	TB patients, *n* = 1116	27.5%
Tarekegne et al.,2016 [52]	Amhara Rgion, Metema Hospital	Retrospective	TB patients, *n* = 2005	20.1%
Teklu et al., 2012 [53]	Sothern Ethiopia, Sedo, institution based	Retrospective	HIV patients, *n* = 459	7.8%
Wondimeneh et al., 2012 [54]	Amhara Region, Gondar university, Hospital based	Cross sectional	HIV patients, *n* = 400,	7.5%
Yassin et al., 2004 [55]	Southern region of Ethiopia., health facilities	Prospective epidemiological	HIV patients, *n* = 261	19%
Degu J. et al. [56]	Southern Ethiopia, Arba Minch Hospital	Cross- sectional	TB patients, *n* = 190	20.1%
Alemie G.A. et al., 2014 [30]	Amhara Region, private health institutions	Cross-sectional	TB patient, *n* = 808	20%
EHNRI, 2014 [57]	Across Ethiopia	Survey in public health facilities	TB patients, *n* = 13,684	20%
EPHI, 2015 [58]	Across Ethiopia	Survey in public health facilities	TB patients, *n* = 7987	17.5%

*EHNRI* Ethiopian Health and Nutrition Research Institite, *EPHI* Ethiopian Public Health Institute.

**Fig. 2:**
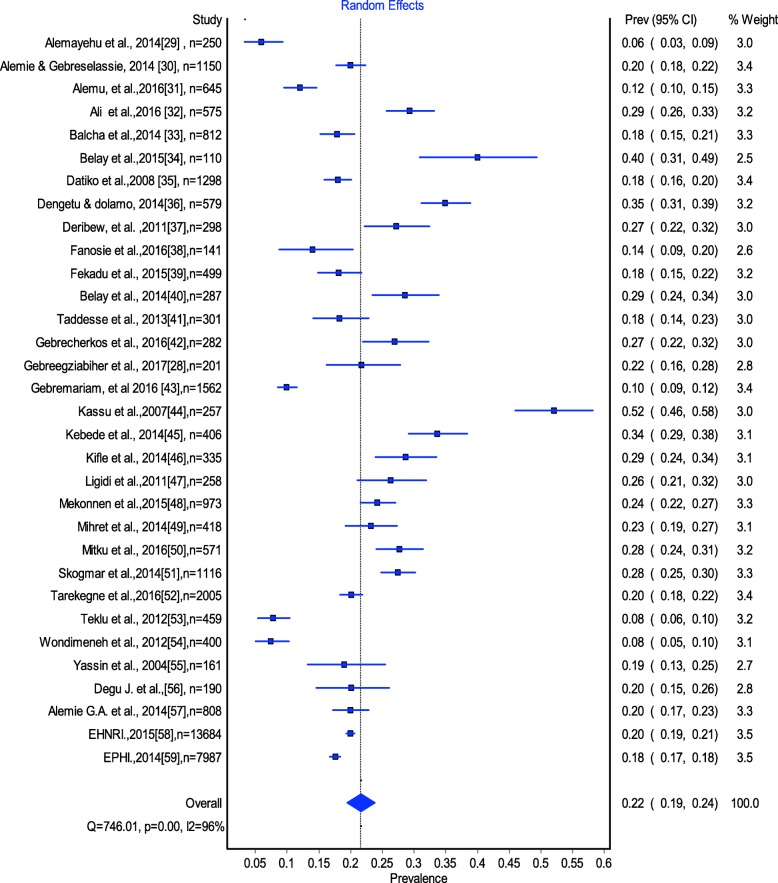
The estimated prevalence of TB-HIV co-infection in Ethiopia: Meta-Analysis

**Fig. 3:**
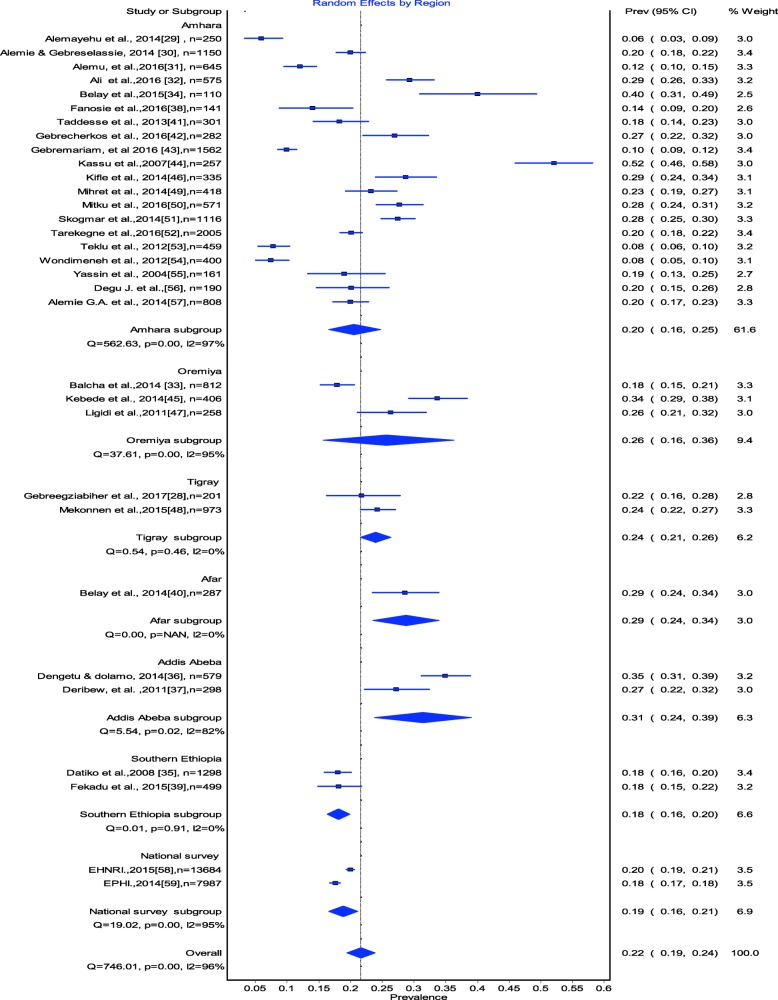
The estimated prevalence of TB-HIV co-infection in Ethiopia by Region Meta-analysis

Estimates of TB-HIV co-infection prevalence ranged from 6 to 52.1% (Fig. 2). The random effects pooled prevalence of TB-HIV co-infection was 22% (95% CI 179–24%) with substantial heterogeneity chi-square (X^2^*)* = 746.0, *p* = 0.0001, (*I*^*2*^ *= 95.85% 95% CI: 94.9–96.6*) (Fig. 2). Prevalence of TB/HIV co-infection was 20% (95% CI 16–24) in the Amhara region, 26% (95% CI 16–36) in the Oromia region, 24% (95% CI 24.21–26.0–) in the Tigray Region, 15% (95% CI 9–21) in Southern Ethiopia, 28% (95% CI 21–36) in Addis Ababa and 34% (95% CI 23–45) in the Afar region (Fig. 3).

As part of our sensitivity analyses, we excluded one large study [28]; estimates of TB-HIV co-infection prevalence did not change.

## Discussion

This systematic review and meta-analysis of TB-HIV co-infection identified 30 studies of 26,746 individuals in Ethiopia. Our pooled results confirmed that the prevalence of TB-HIV co- infection is 22% in Ethiopia. Furthermore, the high prevalence of HIV/AIDS in the general population is related to a higher prevalence of TB-HIV co-infection. This result is almost consistent with one global study [59] but higher than the finding in China (11%) [60], Nigeria (17.5%) [61] and Ghana (18.2%) [62].

According to the subgroup analysis, the highest prevalence was found in the Addis Ababa region (31%), followed by the Afar regional state (29%), whereas the lowest prevalence was found in southern Ethiopia (18%). The substantial difference between the regions might be due to the difference in the socio-cultural and demographic characteristics of the population and associated HIV prevalence [63, 64]. Supporting this, the rank of the regions based on the prevalence of HIV evidenced in the Ethiopian Demographic Health Survey (EDHS) 2016 report [64], is majorly consistent with the rank of the regions in this pooled analysis. This is logically sounding because HIV is the driving factor for TB infection as a result of the immunosuppression.

As Ethiopia is among the high burden countries for TB and HIV, it may be expected to find a high prevalence of TB-HIV co-infection. However, the pooled prevalence of TB-HIV co-infection (22%) is still higher than the WHO estimated figure (8%) in 2017 [65]. The pooled prevalence of TB-HIV co-infection in the current study is bothersome considering the feared complications and emergence of drug resistance; in Ethiopia, the estimated prevalence of MDR-TB is particularly high in previously treated cases (14%) [65]. In addition to this, HIV had been significantly associated with MDR-TB; studies indicate that there was a high rate of MDR-TB among HIV positive individuals; 14% in Addis Ababa [66], 19.4% in the Amhara region [67] and 19.5% in the Oromia region [68]. However, only 70% of those with MDR-TB are thought to be started on second-line treatment and the coverage of ART initiation was 88% in those confirmed to have TB [65]. Moreover, as part of the millennium development goals and now the health sector transformation plan, Ethiopia has been working to reduce HIV and TB associated morbidity and mortalities. Therefore, the pooled prevalence in the current study is still alarming despite the previous efforts. It implicates that efforts are still needed in reducing HIV associated TB which may include prevention, early detection and comprehensive management of cases. As part of the early detection of cases, TB contact household based screening may have a significant input to augment the facility-based screening of TB among HIV positive individuals [69]. In addition to the prevention, early diagnosis helps the early initiation of ART which in turn significantly increases the success rate of TB treatment among HIV patients [70].

TB-HIV co-infection has a great impact on the health outcome of the victims due to accelerated progression. Co-infected individuals have significantly lower years of survival as compared to those HIV positive only [60]. Witnessing this, though the case fatality ratio of TB in Ethiopia is 0.17, the case fatality ratio of TB-HIV together is estimated to be 0.28 [65]. The simultaneous occurrence of the two infections results in different immunological changes, such as the polyclonal activation of HIV harboring lymphocytes and an increased survival of the bacilli inside macrophages [71]. In addition, there is a clear impact on treatment success rate; as compared to TB alone or HIV alone, co-infected patients are more vulnerable to treatment failure and there is a low rate of treatment success [61]. Poor treatment outcome is 2 times higher among TB-HIV patients [48]. Therefore, this TB treatment failure, poor adherence to ART, the progression of the two diseases and the presence of other co-morbidities may significantly facilitate the death of TB-HIV patients [72]. As a limitation, this study did not explain the heterogeneity in terms of differences in the study setting, population or year. In addition, due to differences in the design and population of the studies, this study did not present the analysis of the trends of prevalence over the years.

## Conclusion

Our analysis indicated that the prevalence of TB-HIV co-infection is high in Ethiopia with substantial regional variation. Therefore, an integrated and consistent facility-based as well as community-based effort addressing the prevention, early detection and management of TB and HIV infection should be further strengthened throughout the country to mitigate the double burden disease.

## Abbreviations

AIDS: Acquired Immunodeficiency Syndrome
ART: Antiretroviral Therapy
CDC: Centers for Disease Control
HIV: Human Immunodeficiency Virus
MDR-TB: Multidrug-Resistant Tuberculosis
TB: Tuberculosis
WHO: World Health Organization
